# Some molecular aspects of larval development in *Paralithodes camtschaticus*

**DOI:** 10.1371/journal.pone.0322234

**Published:** 2025-04-29

**Authors:** Alexey V. Boyko, Igor Yu. Dolmatov, Alexander S. Girich, Sergey I. Maslennikov

**Affiliations:** A.V. Zhirmunsky National Scientific Center of Marine Biology, Far Eastern Branch, Russian Academy of Sciences, Vladivostok, Russia; Shanghai Ocean University, CHINA

## Abstract

The transcriptome of the red king crab, *Paralithodes camtschaticus*, was sequenced at four developmental stages: zoea I, zoea IV, glaucothoe, and juveniles. Based on our RNA-seq data and paired-end reads from 112 libraries obtained by other researchers earlier, the transcriptome assembly for *P. camtschaticus* that we obtained has proven to be the most complete of those reported to date. An analysis of enriched processes at different stages has shown, that some of adaptations, e.g., to elevated temperature and hypoxia, do not appear in early larvae. Thus, it is important to maintain optimal conditions for normal larval development and reduce mortality rates. According to the results of the expression profile clustering and transcription factor (TF) search, most TFs are associated with the development of various organs, metamorphosis, and immune responses. The data obtained provide an additional basis for deeper investigation into the mechanisms of the biphasic life cycle in decapods and can be helpful in commercial red king crab stock enhancement programs.

## Introduction

Species of Decapoda have a complex life cycle characterized by complete metamorphosis and multiple developmental stages. These include several long pelagic larval and semibenthic postlarval stages. Some species undergo terrestrialization events, like those in the coconut crab, while others have marine and freshwater stages [1,2]. The variety of habitats used by one taxon or even species makes it possible to study the molecular aspects of adaptations to different conditions at different development stages. Decapods are of great interest as targets of commercial fisheries in China, Russia, South Korea, North Korea, Japan, Canada, and the USA. As a result, populations of the most commercially valuable species have drastically declined [3,4]. Therefore, commercial fisheries for red king crab, blue king crab, Tanner crab, and snow crab have been banned in the Bering Sea and Alaskan waters since the 1990s [5]. For this reason, methods for cultivating these species are being actively studied [5–7].

Unlike a number of other decapod species, it takes about ten years for red king crab to reach the commercial size, which makes its cultivation under artificial conditions commercially unprofitable. A possible solution to the problem is to increase natural stocks through release of hatchery-reared juveniles. Such programs have been implemented by Russia in the Barents Sea, the USA in Alaska, and Japan and Russia in the Sea of Japan [5]. Moreover, rearing of red king crab larvae under controlled conditions significantly increases survival rate to 29–50% vs. 0.7–3% in ocean [5,8]. The causes of such a high mortality in nature are presumably cannibalism, overfishing, egg and larvae predation, diseases, unsuccessful molt, and climate change [5,9,10].

Similarly, the major causes of death of red king crabs during cultivation are cannibalism, unsuccessful molt, and diseases [10,11]. For example, in 1997, an epidemic of *Vibrio* spp. occurred in the Nemuro laboratory, which resulted in the death of 30,000 larvae and a one-season delay in crab production [5]. King crabs have a complex life cycle, with multiple long larval stages, metamorphoses, and molts, which makes crabs vulnerable to pathogens. Crustacean’s pathogens are usually fungi, virus, filamentous bacteria, filamentous diatoms, sessile and saprophytic protistans, and helminths [5]. Although the immune responses to various pathogens have been well studied, the immune-related genes remain poorly known [5,12]. Among these genes, antimicrobial peptides (AMPs), which actually provide multipathogen protection, are of particular interest [13]. To date, a total of 49 AMP candidates have been identified in red king crabs [14]. Despite the high mortality from diseases during development and the importance of AMPs in protection against them, no studies on the expression of the precursor genes of these peptides in red king crab during development have been conducted. Moreover, there is only one publication on changes in gene expression during development [9] and also one on gene expression in a number of organs at the premolt stage in adult red king crabs [15]. However, in the former study [9], the authors did not focus on gene expression in successive stages of red king crab larval development, but rather on the effect of pH and temperature in the prezoeal, juvenile, and adult red king crabs.

In 2021, the first two genomes of blue and red king crabs were published [2,12]. However, the red king crab genome is highly fragmented, including a large proportion of fragmented genes, which makes it difficult to use it for omics research. In our present study, we attempted to obtain the most complete transcriptome and identify differentially expressed genes (DEGs) at the main developmental stages of the red king crab, *Paralithodes camtschaticus*, and the transcription factors (TFs) regulating these genes. We expect the data obtained to become a basis for comparative studies of decapod development, which will eventually increase cultivation efficiency. Along with improving cultivation methods, it is necessary to elucidate the genetic mechanisms of animal resistance to infections. Therefore, we also attempted to identify variations in the expression of AMP precursor genes during development.

## Results and discussion

### *De novo* transcriptome assembly

**Fig 1:**
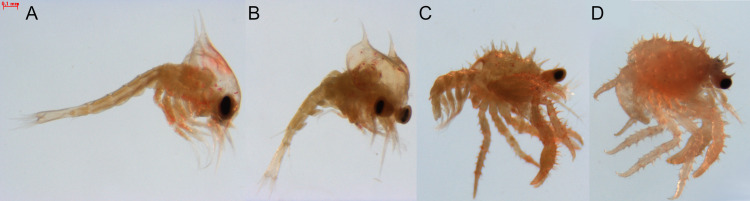
Developmental stages of *P. camtschaticus* used in the study. (**A**) Zoea I, (**B**) zoea IV, (**C**) glaucothoe, and (**D**) first-stage juvenile.

Sequencing of four libraries corresponding to the four developmental stages (Fig 1) of *P. camtschaticus*—zoea I, zoea IV, glaucothoe, and juvenile—resulted in a total of 112.2 million raw paired-end reads. For further assembly, paired-end reads from 112 libraries obtained in previous studies [9] were added to these paired-end reads. After trimming adapters and filtering low-quality and contaminated reads, 94.35% of the paired-end reads were retained, with an average Phred quality score of 35.6. As a result, 1.028 billion filtered paired-end reads with an average read length of 94.8 nucleotides were obtained (S1 Table).

The first stage of assembly in SPAdes resulted in a total of 839,551 contigs. This was unsatisfactory, as the percentage of redundancy of almost identical contigs was high. Therefore, we used the previously described tool HomoloCAP [16] to achieve the final assembly. As a result, we obtained a total of 162,557 contigs and 149,603 non-redundant contigs. Of these, 155,972 contigs had protein-coding sequences (CDSs) with a length of more than 150 bp.

**Fig 2:**
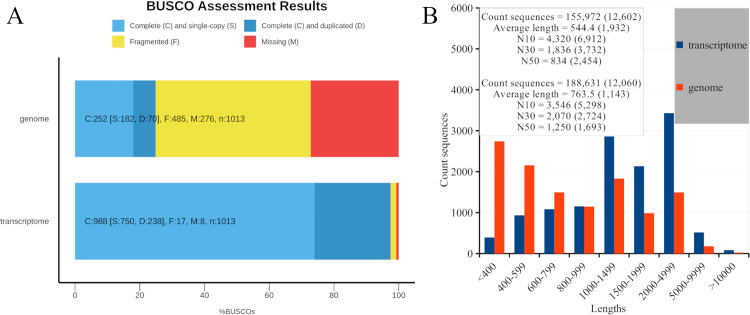
Basic comparison of CDSs from genome and current transcriptome assemblies. (**A**) BUSCO assessment results based on arthropod core genes. (**B**) Length distribution and basic statistical parameters of CDSs that aligned with Chinese mitten crab proteins; values in brackets correspond to CDSs with a length of more than 200 bp with significant BLAST hits in the Chinese mitten crab proteome. For comparative analysis, we used the genome assembly from Veldsman et al. study [2].

Since all the transcriptome assemblies contained more than only protein-coding sequences (CDSs), to achieve a standardized comparison of genome [2] and our transcriptome assemblies, we aligned the predicted protein sequences with those of the Chinese mitten crab, *Eriocheir sinensis* [17]. We then retained only the best matches for each mitten crab protein (S2 and S3 Tables). Our assembly covered 69% of entire proteome of the Chinese mitten crab. Moreover, the genomic assembly included only 46% of mitten crab proteins. It was also supported by BUSCO assessment results that showed numerous missing and fragmented core arthropod genes in the red king crab genome assembly (Fig 2A). Compared to those of the genome assembly, our transcriptome assembly had more contiguous predicted protein sequences that aligned with mitten crab proteins (Fig 2B: see values in brackets). Hence, this assembly can serve as a reference assembly to search for polymorphisms, analyze gene families, etc.

### Differential expression analysis

**Fig 3:**
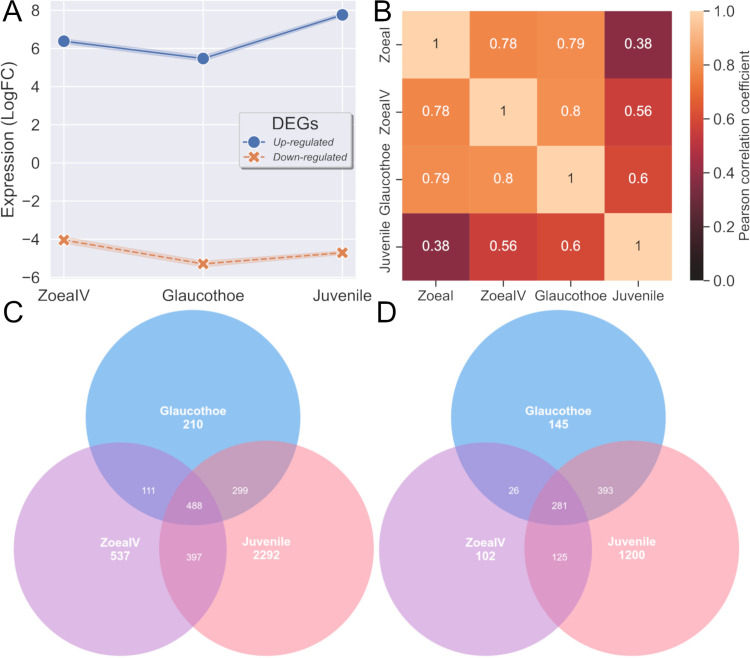
Brief description of results of differential expression analysis. (A) Average LogFC of up- and downregulated DEGs. (B) Correlation map of all RNA-seq samples. (C) Venn diagram of up- and downregulated (D) DEGs.

A search for DEGs revealed a total of 5,909 genes whose expression level significantly changed relative to that in the zoea I stage (S1 File). Of these, 4,640 sequences had significant hits against the NCBI NR database. There were 2,067, 1,953, and 5,475 DEGs at the zoea IV, glaucothoe, and juvenile stages, respectively. There were 845 DEGs common to all three stages. Among the DEGs expressed during the glaucothoe stage, the lowest level of expression was detected (Fig 3A). These observations are strongly consistent with the morphological features of crab development. Zoea I–IV are swimming and planktotrophic larva with high feeding rate [5]. The duration of each of the four zoea stages is approximately 10 days [18]. Glaucothoe is a transitional stage to the benthic juvenile stage. Unlike other stages, it is non-feeding and sedentary. These facts may explain the decrease in gene expression and number of upregulated DEGs.

This observation is also consistent with the number of unique DEGs and the correlation map (Fig 3B and 3C). There was a significant difference between the larval and juvenile stages in the correlation map, with the glaucothoe being in an intermediate position. According to the results of this analysis, the number of positively regulated genes decreased during the transition to glaucothoe and then increased again in juveniles (Fig 3C). The opposite situation is observed for negatively regulated genes (Fig 3D). Judging by the DEGs, the glaucothoe stage was closest to the juvenile stage, rather than to the zoea IV stage (Fig 3C and 3D). This dynamics is likely to be a result of major changes that take place in the work of the genome during the transition to the benthic form, gradual structural complications, and laying down of new tissues and organs.

### Annotation

Annotation of 162,557 contigs by BLAST search against the NCBI protein non-redundant database resulted in identification of 81,178 contigs with significant hits, with 8,654 contigs having only unnamed hits (S4 Table). Hits belonged to 3,984 organisms; more than half of the contigs matched arthropod proteins and only 37% of the contigs matched to decapod proteins (S5 Table). This indicates a rather low representation of decapods in the protein database and, accordingly, scanty available information about the decapod proteins. In addition, there was a high probability of the presence of contaminant sequences. To identify contamination, we compared the bitscore value of sequences matched to non-arthropod proteins in the non-redundant database with those obtained by BLASTp search against the *P. camtschaticus* genome. If the bitscore was lower in the case of the genome, then the sequence was considered contaminant. Thus, we deleted 11,021 contaminant sequences mostly from the phyla Ciliophora, Streptophyta, and Chordata. Even after that, in the final version of the assembly, approximately 11% of the sequences resembled proteins of Ciliophora (S5 Table). However, the level of similarity and the lack of a verified and well-annotated genome in *P. camtschaticus* prevented unambiguous definition of these contigs as a consequence of contamination.

**Fig 4:**
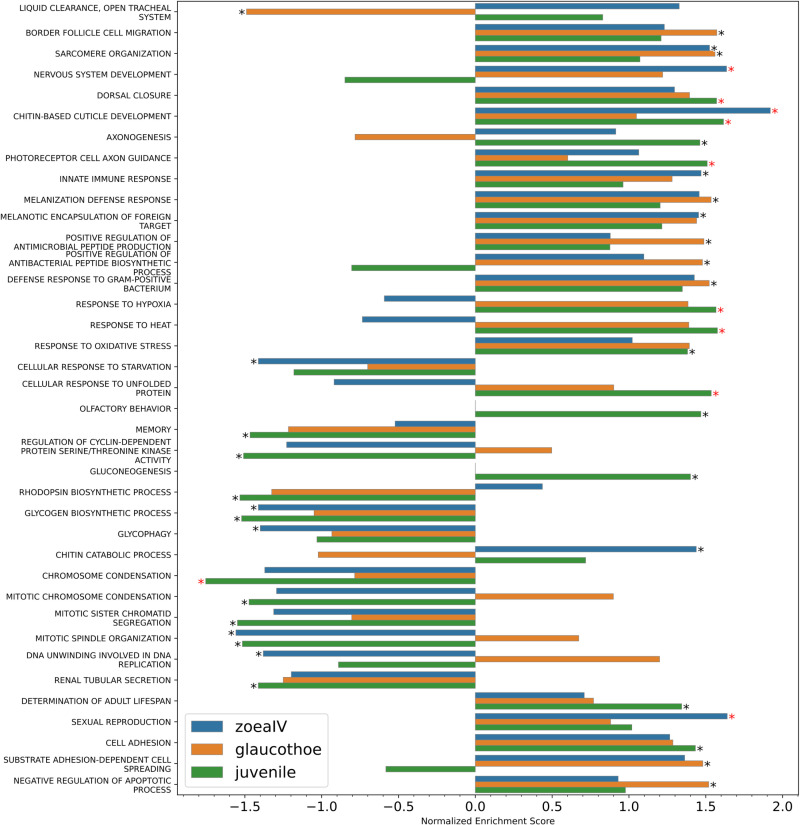
GSEA NES values of significantly enriched processes at three stages of development relative to those at the zoea I stage. Black asterisk indicates a p-value < 0.05; red asterisk, a q-value < 0.25.

During GO annotation, the number of contigs with hits significantly decreased compared to that in BLAST searches against the NCBI database. Of the 7,964 *D. melanogaster* orthologs (S6 Table), only 1,245 exhibited noticeable expression at least at one developmental stage. The significantly enriched processes at different stages of *P. camtschaticus* development are shown in Fig 4 (also S2 File). As mentioned earlier [19], since the GO annotation is based on the functions of proteins of model organisms, GO enrichment analysis provides only a superficial understanding of the processes prevailing at one or another stage of development, the place where these occur, and their functions in *P. camtschaticus*. Nevertheless, this analysis is useful for an overall assessment of gene activity and overall understanding of crab physiology.

Based on the comparison of significantly enriched processes, one can assume that in early larvae of *P. camtschaticus* some adaptation mechanisms do not function, in particular the adaptation to elevated temperature and hypoxia (Fig 4). This conclusion is of great practical significance. Maintaining an optimal temperature and high aeration of water is crucial for the normal development of *P. camtschaticus* larvae and reduction in the mortality rate during cultivation at the zoea I–IV stages [5].

Of interest is the observation that the glaucothoe stage is distinguished by activation of processes related to mitotic cell division. Although larvae do not feed during this period [18], apparently, there is active development and rearrangement of tissues aimed to prepare the body for the settlement and transition to a benthic lifestyle.

It is also worth noting that such processes as “olfactory behavior” and “gluconeogenesis” are activated in juveniles. After settling, crabs switch to an active lifestyle, which is characterized by great physical activity, search for food, and avoidance of predators. In this regard, the need to generate additional energy (gluconeogenesis) and enhance the sense of smell (olfactory behavior) increases.

### TFs searching and clustering of expression profiles

A search for orthologs of *D. melanogaster* transcription factors resulted in identification of 353 TFs (S7 Table). Among them, 22 TFs exhibited significant changes in expression relative to those in the zoea I stage, 18 of which had a “transcription factor” molecular function, as specified in FlyBase. All homologs were also verified against the NR NCBI database.

**Fig 5:**
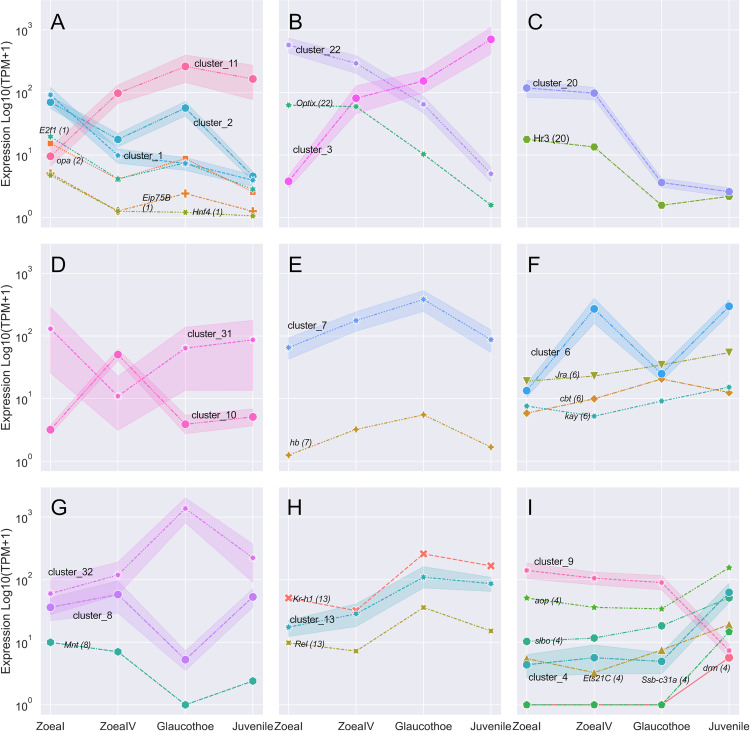
Gene cluster and TF gene expression profiles. (**A**) Clusters 1 and 2 has a positive peak, and cluster 11 has a negative peak at the zoea I stage. (**B**) Clusters 3 and 22, in which the expression levels gradually increased and decreased, respectively. (**C**) Cluster 20 has positive peaks at the zoea I and IV stages. (**D**) Clusters 10 and 31 have positive and negative peaks, respectively, at the zoea IV stage. (**E**) Cluster 7 has positive peaks at the zoea IV and glaucothoe stages. (**F**) Cluster 6 has peaks at the zoea IV and juvenile stages. (**G**) Clusters 32 and 8 have positive and negative peaks, respectively, at the glaucothoe stage. (**H**) Cluster 13 has positive peaks at the glaucothoe and juvenile stages. (**I**) Clusters 4 and 9 have positive and negative peaks, respectively, at the juvenile stage.

Clustering of expression profiles resulted in 71 groups, of which many already contained fewer than 50 sequences. In this regard, clusters with similar expression patterns were manually combined. As a result, 15 clusters were obtained, each of which contained at least 100 sequences, except for cluster 31 with 64 sequences (Fig 5). Of these clusters, nine included TFs.

Apparently, **the zoea I stage** in *P. camtschaticus* was largely characterized by cluster 1 which included 546 genes whose expression peaked at this developmental stage (Fig 5A). The cluster contained GO terms such as “chitin-based cuticle development” and “motor neuron axon guidance”. This cluster contained also three significantly differentially expressed TF genes (*Hnf4*, *Eip75B*, and *E2f1*). These homologs play important roles in the early development of *D. melanogaster*. The hepatocyte nuclear factor 4 (Hnf4) controls the expression of genes involved in glucose and lipid metabolism [20,21]. It is known that the embryonic development of red king crab is lecithotrophic and prezoea larvae contain many yolks [22]. It is likely that the utilization of stored nutrients continues in zoea I because, at this stage, the number of lipid inclusions in tissues decreases compared to that in prezoea. A similar process is regulated by Hnf4 in Xenopus larvae [23]. Moreover, zoea I is the first active feeding larva in red king crab [18]. Hunting requires substantial energy, which is provided by increasing glucose and lipid metabolism. It is also known that HNF4 function in the maturation of the mouse intestinal tissue. It is possible that by the zoea IV stage the intestinal epithelium is already formed in terms of the status of cells and their composition, and therefore the expression level drops. Another possible reason for the drop in expression is interesting: in zebrafish larvae, *hnf4* activity is suppressed in the intestine after microbial colonization [23], which in crabs may end by the zoea IV stage.

The ecdysone-induced protein 75B (Eip75B) regulates key aspects of larval development and metamorphosis *D. melanogaster* [24,25]. The E2F transcription factor 1 (E2f1) is essential for organ growth, development, and metamorphosis via cell cycle regulation [26]. After hatching, crab larvae, like many other arthropods, enter interdependent cycles of molting and growth. This effect is likely associated with the activation of expression of *Eip75B* and *E2f1* in zoea I that are involved in the regulation of molting and organ growth mechanisms at this stage. Eip75B is also involved in the specification of midgut cells, which, given the peak expression of its gene at the zoea I and glaucothoe stages, indirectly indicates the involvement of TF in intestinal development [27]. This is consistent with the changes in midgut at the glaucothoe stage [5]. E2f1 is likely to be involved in metamorphosis more generally through the control of apoptosis, which is why its peak expression is observed at the zoea I and glaucothoe stages [28]. Also, in *Drosophila*, its direct involvement in myogenesis has been shown, which makes this gene important for the crab’s muscle mass gain [29]. Accordingly, this may be important for reproduction, as female crabs perform a kind of test for their partners, during which male crabs must carry the females with their chelipeds.

A less obvious peak at the zoea I stage was associated with cluster 2 that contained a total of 637 genes and the *opa* TF gene. This cluster reflects the processes of this stage, including the tracheal system and midgut development, neurogenesis, and cell differentiation. The odd paired (opa) protein is essential during embryonic segmentation, midgut formation, and adult head morphogenesis [30]. Interestingly, based on the expression profile, the Eip75B and E2f1 TF genes are closer to cluster 2 than to cluster 1, and all of them, including opa, are associated with midgut development [26,27]. Cluster 11, with a negative peak at the zoea I stage, did not have any significantly expressed TFs or enriched processes.

Cluster 22 had a declining expression profile and no significantly enriched processes but one transcription factor, Optix (Fig 5B). The Optix factor is required for the formation of head skeletal elements and eyes in *D. melanogaster* [31]. Obviously, in *P. camtschaticus*, it is involved in the formation and metamorphosis of eyes at the prezoea–zoea I stages and zoea I–glaucothoe stages. Cluster 3 had an inclining expression profile and various processes associated with fatty acid elongation but not with any TFs.

Cluster 20 (Fig 5C) had a peak at the zoea I–IV stages and Hr3 TF. The Hormone receptor 3 (Hr3) is required for developmental progression through early metamorphosis in response to ecdysone [32]. At this time, the inductive function of the Hr3 can be negatively regulated by heterodimerization with Eip75B, which is expressed at the zoea I stage (see cluster 1) [33]. At the same time, Eip75B is a downstream gene for Hr3. Probably, the expression of Hr3 in the zoea I and zoea IV stages is associated with the preparation for metamorphosis or with metamorphosis process itself. However, the cluster had the GO term “wound healing”.

Clusters 10 and 31, with positive and negative peaks at **the zoea IV** stage, respectively, did not have any significantly expressed TFs (Fig 5D). There were clusters 6 and 7 with peaks at the zoea IV stage, the same as at the glaucothoe (7) and juvenile (6) stages (Fig 5E and 5F). Cluster 7 had a peak at the zoea IV and glaucothoe stages. It included 281 genes and no significantly enriched processes. The cluster included hunchback (hb) TF. The hb protein regulates the neural progenitor competence and the body plan formation in *D. melanogaster* [34,35]. TF of *hb* gene is likely to perform similar functions in the development of *P. camtschaticus*. It should also be noted that in fact glaucothoe differs little from the juvenile form, while during the zoea–glaucothoe transition there is a significant change in the body plan, which may explain the peak of *hb* expression at the glaucothoe stage. It is difficult to say anything about the participation of hunchback in the specification of neural cells in this case, since otherwise one could expect the presence of enriched processes associated with this process.

Cluster 6 included the processes of Malpighian tubule morphogenesis and development, glial cell migration, and immune response. The cluster included numerous genes (860) with different expression profiles, but the clustering method could not be used to divide the genes into distinct groups with more precise expression profiles. Thus, in the cluster, there were four genes encoding TFs (*cabut* (*cbt*), *Jun-related antigen* (*Jra*), and *kayak* (*kay*)), whose expression gradually increased or peaked at the glaucothoe stage (*cbt*). These TFs are associated with the development of various systems, including the nervous and respiratory systems, the immune response, metamorphosis, and circadian behavior [36–41]. Thereby, these genes are likely necessary for proper development and timely completion of metamorphosis; therefore, attention should be given to these genes when further studying of larval development. It is difficult to assume anything about the specific functionality of these genes, since their expression profile differs significantly from the cluster profile, making it difficult to rely on the functionality of the cluster genes.

Cluster 32, which exhibited a positive peak in expression at **the glaucothoe stage,** did not contain TFs whose expression significantly changed (Fig 5G). This cluster contains various processes immune response activation, apparently especially important at this stage [42]. For cluster 8, a decrease in expression was recorded at the glaucothoe stage. The GO enrichment analysis revealed only two terms, “fatty acid elongation, saturated fatty acid” and “fatty acid elongation, monounsaturated fatty acid”, which describe metabolic processes. The decrease in the gene expression of this cluster in glaucothoes was likely associated with reduced feeding at that stage. The cluster included a total of 110 sequences and one TF, Mnt. The Mnt protein antagonizes the cell growth-promoting functions of the product encoded by Myc [43]. Notably, *Drosophila* with Mnt and Myc null mutation grow and develop faster then dMyc only mutant [44], which is consistent with the almost complete absence of *myc* expression during zoea IV–glaucothoe stages and the acceleration of growth rates and body mass gain during these stages. Also Mnt null mutation decrease heat tolerance at both normoxia and hypoxia in adult *Drosophila melanogaster* [45]. Thus, the eleven-fold decrease in *mnt* expression at the glaucothoe stage may explain the high mortality during this period. Moreover, given the decrease in the expression of genes associated with fatty acid elongation and elongation of the fatty acid chain in response to a decrease in temperature, the range of normal temperatures for successfully passing the glaucothoe stage decreases at the lower limit. However, it is difficult to imagine the fixation of such a mechanism under selection pressure, and therefore further study of the function of Mnt during larval development in the red king crab is necessary.

In addition, the glaucothoe stage, along with the juvenile stage, was characterized by cluster 13 (Fig 5H) that included 254 genes and two TFs (Rel and Kr-h1). There were three enrichment processes here: “response to starvation”, “metamorphosis”, and “response to ecdysone”. These GO terms were related with the processes occurring at the glaucothoe stage. During this period, the larva does not feed and transforms from planktonic to benthic form [18]. The Relish (Rel) protein regulates antibacterial and antiviral responses, including through activation of AMP precursor genes expression [46–48]. These processes are present in the cluster, but with a low level of significance. However, as will be described below, at the glaucothoe stage the expression of a number of AMP genes is greatly increased. It has also been shown in *Drosophila* that the expression of certain AMP genes is regulated in a developmental stage specific manner [49]. This may indicate a high probability of infections at specific stages of larval development. The Kruppel homolog 1 (Kr-h1) functions as an ecdysone-induced metamorphosis regulator and repressor of neurogenesis in *D. melanogaster* [50]. The latter function of Kr-h1 in *P. camtschaticus* is associated with the inhibition of axonogenesis at the glaucothoe stage (Fig 4). The functioning of this gene as a key regulator of metamorphosis has been studied quite extensively in various species of Arthropoda, and the expression pattern coincides with that obtained in the example of larval development of the mud crab, *Scylla paramamosain* [51].

**The juvenile stage** was characterized by clusters 4 and 9, but the latter had no TFs (Fig 5I). Cluster 4 was the largest one in number of genes and contained 1,824 contigs, being the third of all the detected DEGs. It also contained most TFs (slbo, Ets21C, Ssb-c31a, aop, and drm). GO terms are associated with various aspects of neural system development and morphogenesis, heart and eye development, and immune response. This corresponds to the activation of organogenesis processes occurring after metamorphosis in crabs.

The slow border cells (slbo) protein is involved in epithelial cell migration during the ovarian development in *D. melanogaster* [52]. It is possible that the function of this gene is not limited to ensuring the mobility of border cells during oogenesis, but is generally associated with the regulation of cell migration, which is necessary during the process of metamorphosis. The Ets at 21C (Ets21C) protein possibly controls gene expression in response to infection, oncogene activation, wounding, and aging [53]. Ets21C has been shown to promote the proliferation of intestinal stem cells in the midgut of fruit fly [54]. It has also been shown that this gene does not play a critical role in development and metamorphosis in *Drosophila* under normal conditions, but it is necessary in all cases where rapid cell division and renewal of at least epithelial cell populations are required [53,55]. Thus, it is likely that it is involved in the crab’s midgut transformation, which occurs at the glaucothoe stage. The Single stranded-binding protein c31A (Ssb-c31a) has unknown functions [56]. The anterior open (aop) protein acts downstream of receptor tyrosine kinase signaling to regulate cell fate transitions critical to the development of many tissues, including those of the nervous system, heart, trachea, and eye [57–59]. The drumstick (drm) protein is involved in developmental patterning, cell fate specification, and gut morphogenesis [60,61]. Apparently, aop and drm in *P. camtschaticus* juveniles regulate similar processes.

Thus, there are a number of TFs specific to different stages of crab larval development, but the most interesting in practical terms are TFs associated with the regulation of the immune response (Relish) and growth (Mnt), in particular muscles (E2f1).

### AMPs searching

We found 23 AMP-containing contigs with significant expression changes at some stage of development. These belong to 14 types of peptides: Buforin I, two acipensins, two ubiquicidins, S. scrofa lysozyme, two beta-thymosins, six crustins, two paralithocins, serine peptidase inhibitor, Kazal type 9 (SPINK9), three anti-lipopolysaccharide factors (ALFs), Scolopendin, Yellowfin Tuna GAPDH-related Antimicrobial Peptide (YFGAP), Bovine Pancreatic Trypsin Inhibitor (BPTI), and Skipjack Tuna GAPDH-related AMP (SJGAP) (S8 Table).

**Fig 6:**
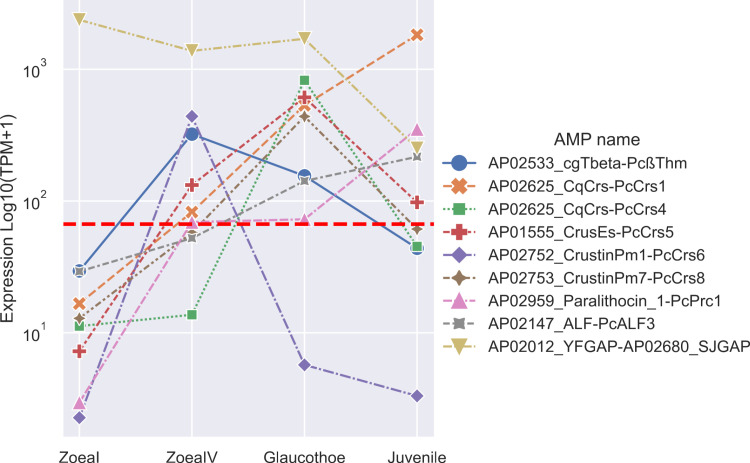
AMP precursor expression profiles. Red line indicates the 67.425 TPM value (third quantile). The peptides are taken from Yakovlev’s study [14] and their names correspond to the APD3 database identifier.

A peak of expression at the zoea I stage was detected for PcBuf2, PcPrc4, Scolopendin, YGFAP, and SJGAP. A peak of expression at the zoea IV stage was detected for PcLys2, PcßThm, PcCrs6, and PcKaz1. PcßThm (other contig), PcCrs4, PcCrs5, PcCrs8, and PcALF2 showed a peak of expression at glaucothoe stage. Most of precursors had peaks at juvenile stage: PcAcp9, PcAcp5, PcUbi1, PcCrs1, PcCrs7, PcPrc1, PcALF1, PcALF3, and BPTI. Peptides with peak expression at certain stage are not grouped on the basis of activity against a particular pathogen type. Therefore, it is difficult to draw any conclusions about the most critical diseases at a particular stage of development. However, most precursors have a relatively low expression level compared to the most highly expressed precursors. Most likely, the concentration of the peptide should be high enough to perform the protective function. In this regard, it makes sense to consider only precursors with the highest expression levels. Thus, if we consider only peptides with a TPM value greater than the third quantile (75%), it turns out that at the zoea I stage there is only one contig left, containing the putative peptides YFGAP and SJGAP (Fig 6). However, this precursor also has high expression levels at other stages. Since these AMPs exert a bacteriostatic effect [62,63], it can be assumed that they are necessary for the control of microbial colonization of the crab intestine. It is also likely that the set of peptides changes depending on the stage, which is especially evident for the glaucothoe stage (PcCrs4, PcCrs5, and PcCrs8). Apparently, this is associated with different pathogens that affect different stages of development. Although there is information on the expression of different peptides at specific developmental stages in the shrimp, Litopenaeus vannamei [64], and *Drosophila* [49], it is difficult to explain this difference by the specifity of AMPs to a particular pathogen without studying changes in the intestinal microbiota and typical diseases at specific developmental stages. For example, different classes of crustins, although they have some difference in activity against Gram-positive or Gram-negative bacteria, overlap in many ways.

## Materials and methods

### Ethical statement

Ethical approval from the Commission on Biomedical Ethics of the NSCMB FEB RAS for procedures on decapod crustaceans is not required in the Russian Federation since our work did not involve vertebrate animals. However, our protocols complied with standard practices including collecting organs and sacrificing animals using cold anesthesia.

### Animals

In December 2021, female red king crabs, *Paralithodes camtschaticus* (Tilesius, 1815), were captured by SCUBA divers in Peter the Great Bay, Sea of Japan. The animals were kept in 4 m^3^ tanks with running aerated seawater. Recirculating water undergoes mechanical filtration and UV disinfection and is under thermostatic control to maintain the required water temperature. The water temperature was 1–4^o^ C. After the larval hatching, the females were released back into the sea. Larvae were kept in the same tanks up to settlement. The larvae were kept at a temperature of 7–9^o^ C and fed nauplii of *Artemia salina*. The housing conditions and the timing of each stage were described more in detail by Gevorgyan and Kovatcheva [8,65].

### Sample collection and RNA extraction

For the analysis, we used zoea I, zoea IV, and glaucothoe larvae and the first juvenile stage. A total of approximately 30 larvae from each of the stages were pooled for RNA extraction and library preparation. For the juvenile library, five individuals were pooled. The experiment was carried out without replicates. Before isolating total RNA, the samples were precipitated in sterile seawater. Homogenization was carried out with TRIzol and metal balls on a TissueLyser LT homogenizer (Qiagen, Germany). Total RNA was isolated by phenol–chloroform extraction [66].

### Transcriptome sequencing

After testing the total RNA on an Agilent TapeStation (Agilent, USA), we prepared the libraries using a TruSeq Stranded mRNA Library Prep Kit (Illumina, USA), and fragments with a length of 200–450 bp, including adapters, were selected. After testing the quality on an Agilent TapeStation, paired-end sequencing (2×100) was performed on an Illumina HiSeq 2500 sequencer. The raw reads were uploaded to the NCBI database under the BioProject accession number PRJNA799484.

### *De novo* transcriptome assembly

To obtain the most complete and accurate transcriptome assembly, in addition to the four paired-end read libraries that had we obtained, we also used the currently available data which included 112 raw Illumina paired-end read libraries (S1 Table) from previous studies [9]. The raw reads in the FASTQ format were processed using Trimmomatic v0.36 [67] with the “LEADING:20 TRAILING:20 SLIDINGWINDOW:5:20 AVGQUAL:25 MINLEN:25” parameters to obtain clean reads by removing those containing adapter sequences, poly-N sequences, or low-quality bases. The clean reads were subsequently classified using the Kraken v2.1.2 [68] software with a custom database that contained archaeal, bacterial, viral, plasmid, human, UniVec Core, protozoan, and fungal sequences as well as rRNA from SILVA v138.1 and *Paralithodes* rRNA from NCBI (the sequences were downloaded on June 23, 2021). The confidence threshold was determined using a series of values ranging from 0.0 to 0.8 and by plotting the count of reads per unique taxonomy rank.

All unclassified paired reads were *de novo* assembled using SPAdes v3.15.3 [69] with k-mer lengths of 33 and 49. Among the obtained contigs, coding sequences (CDSs) with a minimum length of 50 aa were extracted using TransDecoder v5.5.0 (http://transdecoder.github.io/). The code of TransDecoder was modified in such a way that the stop-codon at the beginning of the sequence, but not the “ATG” (Met), was an indicator of the beginning of the CDS. All CDSs were verified using BLASTp v2.12.0 [70] against the Decapoda proteins of the NCBI non-redundant database (downloaded on December 19, 2021), as is described in the manual to TransDecoder.

Then, all the obtained CDSs were subjected to the assembly stage in HomoloCAP as described previously [16] with slight modifications. The obtained assembly was filtered to remove obvious contaminants that were found matching non-arthropod proteins. The assembly was subsequently filtered according to the NCBI TSA submission guide. The resulting assembly was uploaded to the NCBI database under the TSA accession number GKTP00000000.

To compare the genome [2] and out transcriptome assemblies, we used only CDSs that had significant BLAST hits in the Chinese mitten crab (*Eriocheir sinensis*) proteome. Also, an analysis of the completeness of core genes was performed for both, genomic and transcriptomic, assemblies using BUSCO v5.2.1 [71] in the “protein” mode with a Arthropoda v10 dataset.

### Differential expression analysis

To identify DEGs, a standard pipeline from the Trinity v2.8.4 software [72] was used; the number of mapped reads was calculated in RSEM v1.3.1 [73]; paired-end read alignment was performed in Bowtie v2.4.4 [74]; the following parameters were added to the default ones: “--nofw --no-mixed --no-discordant --no-contain --gbar 1000 -N 1 --end-to-end -k 20 -q --maxins 1000”. The Trinity’s standard procedure for detecting significant changes in the expression was modified. Thus, only sequences with more than 3.69 TPM in at least one stage (median value) were included in the analysis; also, a zero number of mapped reads was allowed at any stage. After this filtration, differential expression was evaluated for the sequences in edgeR v3.38.4 [75] with a specified level of dispersion of 0.1; the DEGs whose absolute value of expression level was four times as high at each of the stages as that at the zoea I stage (|LogFC|≥2) and whose Padj value was lower than 0.01 were considered actual.

### Annotation

Annotation was carried out against several protein databases with a standard e-value of 1×10^-6^ for BLASTx. Basic annotation was performed against the NCBI NR database; GO annotation was carried out against the FlyBase database v6.46; orthologs of *D. melanogaster* proteins were identified using a custom Python script that implements a modified reciprocal method for finding the best hit, as described in the article [19]. The annotation for identifying transcription factors (TFs) and the comparative analysis of assemblies were based on found orthologs and predicted TFs from the FlyMine v53 database. To identify the most vivid TFs in terms of expression, only the TFs identified as DEGs, were considered.

The GO enrichment analysis was carried out using the GSEA v4.1 [76] software with parameters “-collapse No_Collapse -mode Max_probe -norm meandiv -nperm 1000 -rnd_seed 1639200481082 -set_max 200 -set_min 5” and a custom gene set created from the FlyBase gene ontology annotation file in accordance with the EnrichmentMap protocol for RNA-seq data (S2 File). In the case of cluster analysis, the test set was the cluster. The rank of a gene had a value of 1 or 0, depending on whether the gene was in the cluster or not.

### Clustering by expression profiles

For clustering, we used only transcripts with significant variations in expression. Clustering was carried out with an 80% threshold of profile matching, using scripts from the Trinity package. Then the clusters similar in profile were manually combined into a single cluster. Eventually, only the clusters with more than 100 sequences were retained.

### AMP searching

For searching of AMPs, we used 49 contigs with previously predicted AMPs of red king crab [14]. We aligned these AMP-containing contigs to our transcriptome assembly using BLASTp. We then extracted the top 10 bitscore matches for each contig and retained those with the fewest substitutions in the peptide region. The obtained AMPs were additionally confirmed by the AMP prediction tool of the CAMPR4 database [77]. We left only peptides with significant changes in expression at any stage.

## Supporting information

S1 Table The basic features of analyzed illumina paired-end libraries after filtration. (XLS)

S2 Table Results of BLASTp search of transcriptome best hits against Chinese mitten crab proteome. (XLS)

S3 Table Results of BLASTp search of genome best hits against Chinese mitten crab proteome. (XLS)

S4 Table Results of BLASTp search of best hits against NCBI protein non-redundant database. (XLS)

S5 Table Taxonomy report of the best hits at the NCBI protein non-redundant database. (XLS)

S6 Table List of the orthologs of *D. melanogaster* proteins. (XLS)

S7 Table List of the putative orthologs of *D. melanogaster* transcription factors. (XLS)

S8 Table List of the putative precursors of antimicrobial peptides with CAMP RF probability, LogFC, FDR, and TPM values. (XLS)

S1 File Files with mapped events, TPM values, and edgeR results of the evaluation of differential expression at third developmental stages relative to the zoea I sample. (ZIP)

S2 File Data files needed for GSEA analysis and creation of Fig 4. (ZIP)
